# LncRNA GAS5 positively regulates IL‐10 expression in patients with generalized myasthenia gravis

**DOI:** 10.1002/brb3.2457

**Published:** 2021-12-22

**Authors:** Shuangshuang Peng, Yangping Huang

**Affiliations:** ^1^ Department of Neurology Taizhou First People's Hospital Taizhou City P. R. China

**Keywords:** IL‐10, lncRNA GAS5, myasthenia gravis, peripheral blood mononuclear cells

## Abstract

**Introduction:**

LncRNA growth arrest‐specific transcript 5 (GAS5) has been proven to be involved in autoimmune diseases. Rheumatoid arthritis is a type of autoimmune disease that may affect myasthenia gravis (MG) patients. However, its direct role in MG is unknown.

**Methods:**

Our study included 62 generalized MG patients. GAS5 expression was analyzed with real‐time quantitative RT‐PCR (qRT‐PCR). The interaction between GAS5 and interleukin 10 (IL‐10) was explored in overexpressed cells using real time quantitative polymerase chain reactions (RT‐qPCRs) and western blot. The correlation of GAS5 and IL‐10 was analyzed using Pearson's correlation analysis. The diagnostic value of GAS5 for MG was analyzed using receiver operating characteristic (ROC) curve analysis.

**Results:**

GAS5 and IL‐10 mRNA levels in peripheral blood mononuclear cells (PBMCs) were significantly lower in MG patients than healthy controls. Downregulated GAS5 effectively distinguished MG patients from healthy controls. GAS5 expression was positively correlated with IL‐10 expression in both MG patients and healthy controls. GAS5 overexpression significantly upregulated IL‐10 expression in PBMCs derived from both MG patients and healthy controls.

**Conclusion:**

LncRNA GAS5 may improve generalized MG by positively regulating IL‐10 expression.

## Graphical abstract

GAS5 is downregulated in PBMCs of MG patients. GAS5 may upregulate the expression of IL‐10 to improve MG conditions.

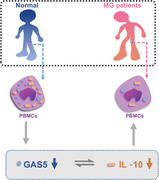

## 1 INTRODUCTION

Myasthenia gravis (MG) is an autoimmune disease characterized by fatigue and muscle weakness. It is a type of B‐cell mediated disorder affecting about 1 out of 50,000 people (Gilhus & Verschuuren, 2015; McGrogan et al., 2010). MG is frequently associated with thymic pathologies, which may affect the production of anti‐AchR antibodies (Gilhus & Verschuuren, 2015; McGrogan et al., 2010). Anti‐AchR antibodies are detected in most MG patients. The major function of these antibodies is to block the communication between muscle cells and nerves (Gilhus & Verschuuren, 2015; McGrogan et al., 2010). MG occurrence is also closely correlated with antibodies directed against muscle‐specific kinase (MUSK), lipoprotein‐related protein 4 (LRP4), or acetylcholine receptor (Gilhus & Verschuuren, 2015). The prognosis of MG is generally good as long as optimum immunosuppressive, symptomatic, and supportive treatments are performed based on subtypes (Mao et al., 2010), which are determined according to serum antibodies screening and observation of typical clinical features. However, long‐term drug treatment and individual treatment strategies are not affordable for most patients (Gilhus & Verschuuren, 2015).

The onset and development of MG is a complex process with multiple molecular players involved (Avidan et al., 2014). Noncoding RNAs (ncRNAs) are a group of RNA transcribed from the human genome. ncRNAs encode no protein, but they play pivotal roles in both physiological processes and pathological changes in the human body (Esteller, 2011). Studies on the involvement of ncRNAs in MG are mostly focused on miRNAs (Punga et al., 2014; Zhang et al., 2016), while the functionality of long noncoding RNA (lncRNA) in this disease is largely unknown. lncRNA growth arrest‐specific transcript 5 (GAS5) has been proven to be involved in rheumatoid arthritis (Yang et al., 2021). MG is closely correlated with allergic diseases, and GAS5 is involved in allergic rhinitis (Song et al., 2021; Yeh et al., 2015). Our preliminary deep sequencing data revealed that GAS5 was downregulated in MG patients and positively correlated with interleukin 10 (IL‐10) (data not shown). It has been well established that increased IL‐10 production is associated with the production of anti‐AchR antibodies and treatment outcomes in MG patients (Zhang et al., 2016). Therefore, this study was carried out to investigate the involvement of GAS5 in MG and its interaction with IL‐10 (Sheng et al., 2015).

## 2 METHODS

### 2.1 Study population

Our study included 62 generalized MG patients. These patients were diagnosed and treated in Taizhou First People's Hospital from January 2015 to January 2018. The diagnostic criteria were (1) patients with serum antiacetylcholine receptor antibodies (AchR Ab); (2) patients with fluctuating weakness of voluntary muscles; and (3) significant improvements were achieved after cholinesterase inhibitor injection and decremental pattern on repetitive nerve stimulation.

Seropositive for anti‐AchR antibodies was observed in all patients. Among these patients, 12 patients showed thymic abnormalities. No other obvious autoimmune diseases were diagnosed in these patients. Patients with other severe diseases were excluded. None of the patients received immunomodulatory drugs within 6 months before admission.

These patients included 35 males and 27 females aged 38–72 years, with a mean age of 53.1 ± 7.4 years. At the same time, a total of 41 healthy people with no inflammation were also included to serve as the control group. The control group included 24 males and 17 females aged 35–69 years, with a mean age of 52.6 ± 7.1 years. No significant differences in age and gender were found between the patients and controls. Ethics Committee of Taizhou First People's Hospital approved the study, and all participants signed informed consent. Table 1 shows the clinical data of both patients and controls.

**TABLE 1:** Clinical data of both patients and controls

	Control	MG
	(*n* = 41)	(*n* = 62)
Gender, M/F	24/17	35/27
Age (years)	53.1 ± 7.4	52.6 ± 7.1
Age at onset (years)	–	41.5 ± 7.1
Anti‐AchR^+^ (%)	–	62/62 (100)
Anti‐MUSK^+^ (%)	–	0 (0)
Thymoma (%)	–	0 (0)
QMG score		19.4 ± 3.4
MG II		44.9 ± 5.9

Abbreviations: MG, myasthenia gravis; MUSK, muscle‐specific kinase; QMG, quantitative myasthenia gravis.

### 2.2 Blood extraction and peripheral blood mononuclear cell isolation

Venous blood (10 ml) was extracted from participants prior to treatments. Blood samples were transferred to heparinized vacuum tubes, centrifuged for 15 min at 3000 rpm, and used to isolate peripheral blood mononuclear cells (PBMCs) through Ficoll gradient separation using lymphocyte isolation agents.

### 2.3 Real‐time quantitative RT‐PCR

Total RNA was extracted from PBMCs using TRIzol reagent (Invitrogen, USA) and reverse transcribed into cDNA using MMLV reverse transcriptase (Epicentre). PCR reaction systems were prepared using SYBR Green kit (Thermo Fisher Scientific Inc.), and PCR reactions were performed on an Applied Biosystems 7900 Fast Real‐Time PCR system. PCR reaction conditions were 95°C for 1 min followed by 40 cycles of 95°C for 12 s and 58°C for 35 s. Primers used in PCR conditions were as follows: 5′‐CCATGGATGACTTGCTTGGG‐3′ and 5′‐TGCATGCTTGCTTGTTGTGG‐3′ for GAS5; 5′‐GCCTAACATGCTTCGAGATC‐3′ and 5′‐TGATGTCTGGGTCTTGGTTC −3′ for IL‐10; and 5′‐GACCTCTATGCCAACACAGT‐3′ (forward) and 5′‐AGTACTT GCGCTCAGGAGGA‐3′ (reverse) for β‐actin. Ct values were processed using the 2^−ΔΔ^ *^Ct^* method, and GAS5 expression was normalized to β‐actin.

### 2.4 Cell culture and transfection

PBMCs were cultured in a 96‐well plate containing RPMI‐1640 medium in an incubator with 95% humidity and 5% CO_2_ at 37°C. PCR reactions were performed to obtain the full‐length GAS5 cDNA fragment containing *EcoR I* cutting site on both sides. This fragment was inserted into *ECOR I* linearized pIRSE2‐EGFP vector (Clontech, Palo Alto, CA, USA) to construct the GAS5 expression vector. Note that 10 nM vectors were transfected into 5 × 10^5^ PBMC cells using Lipofectamine 2000 reagent (11668‐019; Invitrogen). After 6 h incubation with transfection mixture, cells were washed with fresh medium and cultured in fresh medium prior to the subsequent assays. Transfection of the empty vector was performed to serve as the negative control.

### 2.5 Western blot

After transfection, total protein was extracted from PBMCs using RIPA solution (Thermo Fisher Scientific). Protein concentration was measured using bicinchoninic acid assays. Protein samples were mixed with loading buffer, denatured, separated by electrophoresis after loaded onto 10% SDS‐PAGE gels with 20 μg per lane, and transferred onto polyvinylidene fluoride membranes. The membranes were blocked with 5% skimmed milk for 1 h at 25°C and incubated with rabbit antihuman primary antibodies against IL‐10 (34843, 1:1500; Abcam) and GAPDH (ab9485, 1: 1400, Abcam) at 4°C overnight. Membranes were then washed with tris buffered saline Tween‐20 (0.3% Tween) and incubated with an horseradish peroxidase‐labeled secondary antibody (1:1000, MBS435036, MyBioSource) for 3 h at room temperature. Signals were developed using ECL (Sigma‐Aldrich, USA), and the grey value of IL‐10 band was normalized to that of GAPDH using Image J software.

### 2.6 Statistical analysis

Graphpad Prism 6 software was used for all statistical analyses. Data with normal distribution were expressed as mean ± SD. Differences between the two groups were and compared using t test and among multiple groups were compared using one‐way ANOVA test, then the LSD‐t method was used for two‐by‐two comparison. Pearson's correlation coefficient was applied to analyze correlations. Receiver operating characteristic (ROC) curve analysis was applied to analyze the diagnostic value of GAS5 for MG. The 62 patients were divided into high and low GAS5/IL‐10 groups (*n* = 31). Associations between GAS5/IL‐10 and patients’ clinical data were analyzed with chi‐squared tests. *p* < .05 was considered statistically significant.

## 3 RESULTS

### 3.1 Comparison of GAS5 and IL‐10 mRNA expression in PBMCs derived from MG patients and healthy controls

**FIGURE 1:**
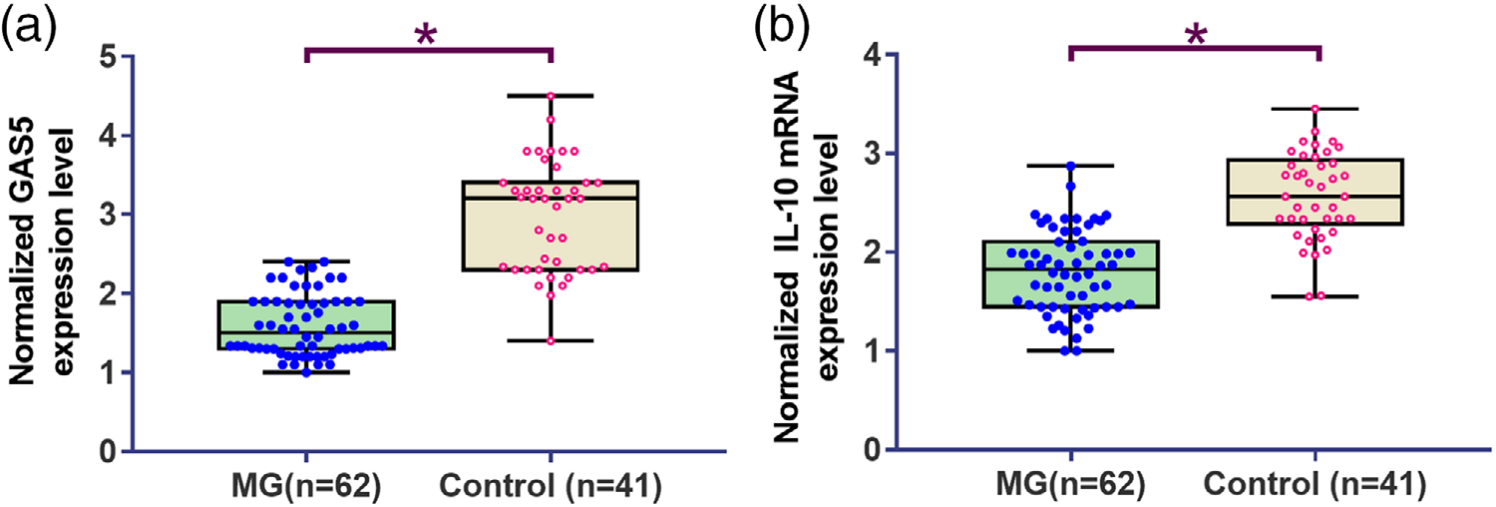
Comparison of growth arrest‐specific transcript 5 (GAS5) and interleukin 10 (IL‐10) mRNA expressions in peripheral blood mononuclear cells (PBMCs) derived from myasthenia gravis (MG) patients and healthy controls. Normalized GAS5 (a) and IL‐10 (b) mRNA levels in PBMCs derived from MG patients and healthy controls. Each dot represents the average value of three technical replicates of real time quantitative polymerase chain reactions (RT‐qPCRs). *Note*: *p < .05

GAS5 and IL‐10 mRNA levels in PBMCs derived from MG patients and healthy controls were detected by qRT‐PCR. As shown in Figure 1a, GAS5 expression levels were significantly lower in MG patients than in healthy controls (*p* < .05). In addition, IL‐10 mRNA levels were also significantly lower in MG patients than in healthy controls (*p* < .05, Figure 1b). Chi‐squared test analysis showed that GAS5 levels were closely correlated with quantitative myasthenia gravis (QMG) score, MGII, and mean anti‐AChR titer, but not with age at onset and thymic abnormalities (Tables 1 and 2).

**TABLE 2:** Associations between GAS5/IL‐10 and patients’ clinical data

		GAS5				IL‐10			
	Cases	High (*n* = 31)	Low (*n* = 31)	Chi‐squared test	*p*	High (*n* = 31)	Low (*n* = 31)	Chi‐square test	*p*
Age at onset (years)									
>42	32	15	17	0.26	.61	18	14	1.03	.31
≤42	30	16	14			13	17		
QMG score									
>20	31	10	21	7,81	.005	21	10	7,81	.005
≤20	31	21	10			10	21		
MG II									
>45	29	10	19	5.25	.02	20	9	7.84	.005
≤45	33	21	12			11	22		
Thymic abnormalities									
Yes	12	7	5	0.41	.52	5	7	0.41	.52
No	50	24	26			26	24		
Mean anti‐AChR titer (10^9^ M)									
>70	34	10	24	12.76	.0003	23	11	9.38	.002
≤70	28	21	7			8	20		

Abbreviations: IL‐10, interleukin 10; MG, myasthenia gravis; QMG, quantitative myasthenia gravis.

### 3.2 Diagnostic value of GAS5 expression in PBMCs for MG

**FIGURE 2:**
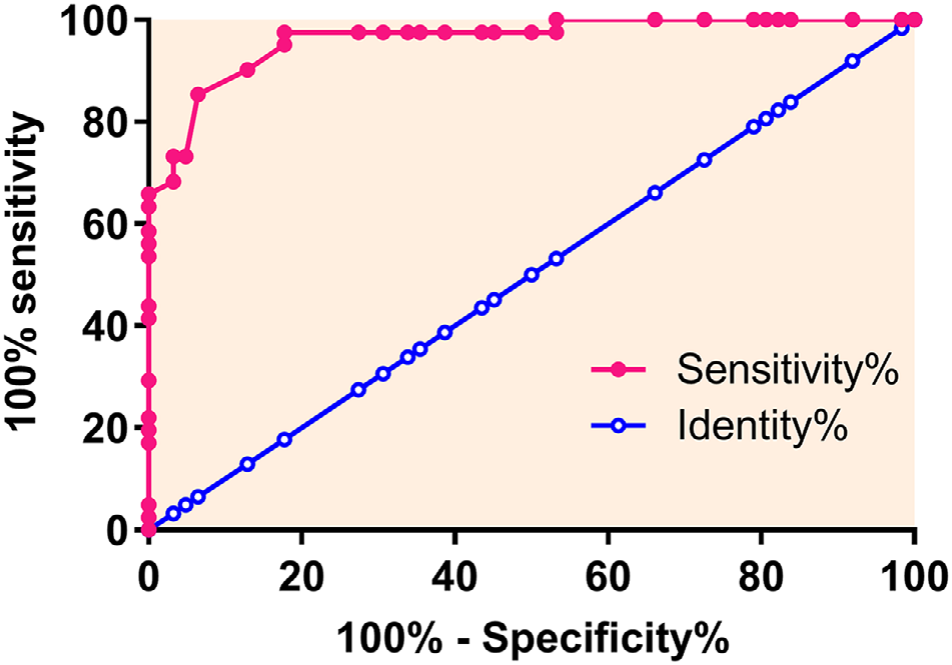
Receiver operating characteristic (ROC) curve analysis of the diagnostic value of growth arrest‐specific transcript 5 (GAS5) expressions in peripheral blood mononuclear cells (PBMCs) for myasthenia gravis (MG). In this analysis, MG patients (*n* = 62) were true positive cases and healthy controls (*n* = 41) were true negative cases

ROC curve analysis was performed to evaluate the diagnostic value of GAS5 expression in PBMCs for MG. As shown in Figure 2, the area under the curve was 0.8969, with a standard error of 0.03517 and a 95% confidence interval of 0.8280 to 0.9659 (*p* < .001). Therefore, GAS5 expression may assist the diagnosis of MG.

### 3.3 Correlation between GAS5 and IL‐10 mRNA expression in PBMCs derived from MG patients and healthy controls

**FIGURE 3:**
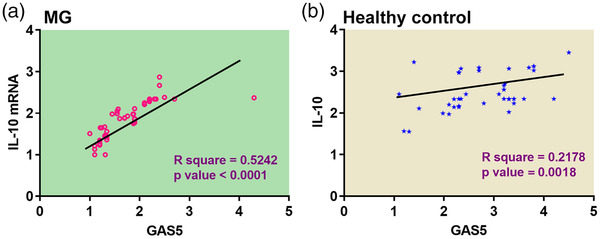
Correlation between growth arrest‐specific transcript 5 (GAS5) and interleukin 10 (IL‐10) mRNA expressions in peripheral blood mononuclear cells (PBMCs) derived from myasthenia gravis (MG) patients and healthy controls. Shown are the results of Pearson's correlation analysis on the correlation between GAS5 and IL‐10 mRNA expression in PBMCs derived from MG patients (a) and healthy controls (b). Average values of three technical replicates were used in correlation analysis

Pearson's correlation analysis was performed to analyze the relationship between GAS5 and IL‐10 mRNA expression in PBMCs derived from MG patients and healthy controls (Figure 3). It was found that GAS5 expression was significantly and positively correlated with IL‐10 mRNA expression in both MG patients (*R* ^2 ^= 0.5254, *p* < .001) and healthy controls (*R* ^2 ^= 0.2178, *p* = .0018). However, the positive correlation between GAS5 and L‐10 mRNA expression was more substantial in MG patients than in healthy controls.

### 3.4 Comparison of GAS5 expression in PBMCs derived from MG patients before and after treatment

**FIGURE 4:**
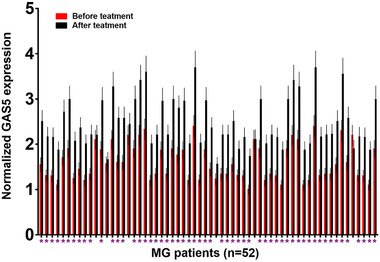
Comparison of growth arrest‐specific transcript 5 (GAS5) expression in peripheral blood mononuclear cells (PBMCs) derived from myasthenia gravis (MG) patients before and after treatment. Real time quantitative polymerase chain reactions (RT‐qPCRs) were performed in three technical replicates. Data from three technical replicates were compared. *Note*: *p < .05

GAS5 expression in PBMCs derived from MG patients was also detected on the day of discharge. As shown in Figure 4, compared with pretreatment levels, a significantly increased GAS5 level was found in 56 out of 62 MG patients (*p* < .05), accounting for 90.3%.

### 3.5 Effects of GAS5 overexpression on IL‐10 protein expression in PBMCs derived from MG patients and healthy controls

**FIGURE 5:**
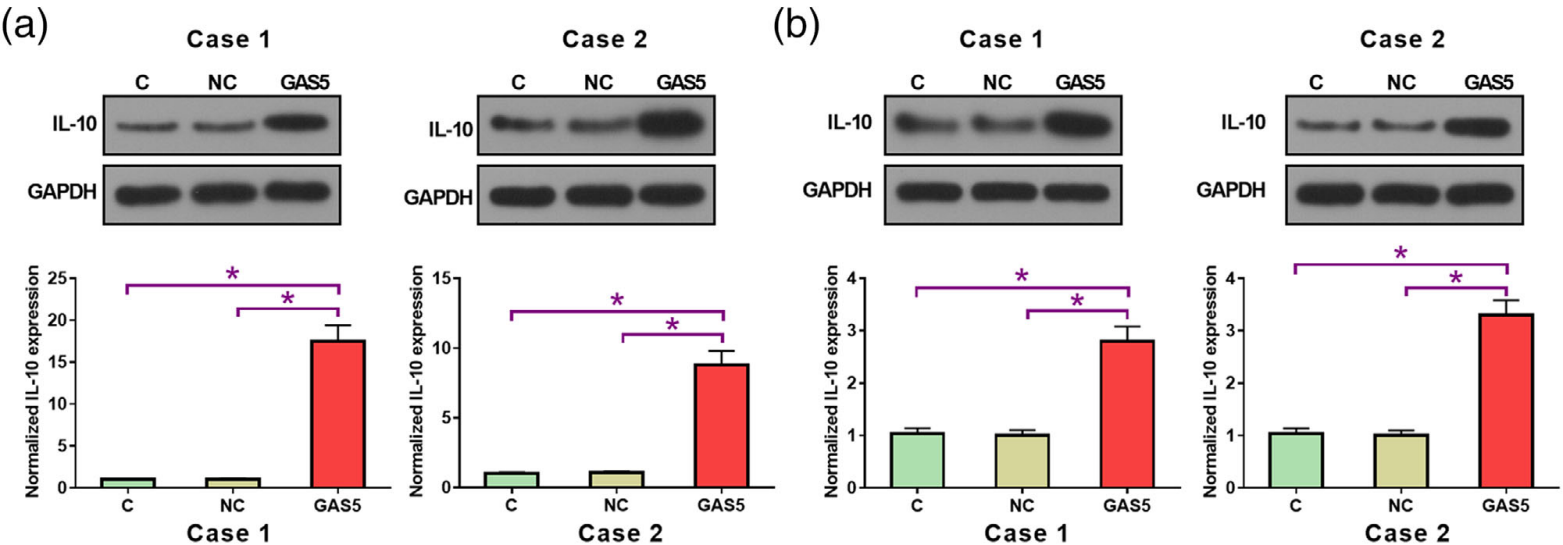
Effects of growth arrest‐specific transcript 5 (GAS5) overexpression on interleukin 10 (IL‐10) protein expression in peripheral blood mononuclear cells (PBMCs) derived from myasthenia gravis (MG) patients and healthy controls. Shown are IL‐10 protein levels in two cases of PBMCs derived from MG patients (a) and 2 cases of PBMCs derived from healthy controls (b). Each experiment was performed in three biological replicates. Data from these three replicates were compared. Abbreviations: C, control cells without transfections; NC, negative control cells transfected with empty vector. *Note*: *p < .05

The positive correlation between GAS5 and IL‐10 expression indicated a potential interaction between them in MG. To further investigate this possible interaction, the GAS5 expression vector was transfected in two cases of PBMCs derived from MG patients and two cases of PBMCs derived from healthy controls. A >200% increase in GAS5 overexpression in MG patients was confirmed by qRT‐PCR. As shown in Figure 5, IL‐10 expression was significantly upregulated in two cases of PBMCs derived from MG patients (Figure 5a, *p* < .05) than in two cases of PBMCs derived from healthy controls (Figure 5b, *p* < .05) after GAS5 expression vector transfection.

## 4 DISCUSSION

The main finding of our study is that GAS5 a well‐established tumor suppression lncRNA in different types of human malignancies (Cao et al., 2014; Liu et al., 2015) may also participate in MG. We also provided evidence that GAS5 could upregulate IL‐10 level in PMBCs, which in turn improved MG.

Although a recent study has observed that the development of MG is accompanied by changes in expression patterns of a larger set of lncRNAs (Luo et al., 2015), clinical trials on the involvement of a specific lncRNA in MG are lacking. Increased risk for complicating autoimmune diseases, such as systemic lupus erythematosus, autoimmune thyroid disease, and rheumatoid arthritis was observed in patients with MG (Nacu et al., 2015), indicating the existence of common pathological changes shared by these autoimmune disorders. It has been reported that lncRNA GAS5 is downregulated in rheumatoid arthritis and osteoarthritis (Yang et al., 2021; Zhang et al., 2021), and its downregulation contributes to the disease progression. In our study, downregulated GAS5 expression was observed in PBMCs of MG patients compared with healthy controls, indicating the involvement of GAS5 expression inhibition in this disease. Comparison of GAS5 expression levels in MG patients and healthy controls showed that GAS5 expression levels were significantly increased after proper treatment (cholinesterase inhibitor treatment), indicating that GAS5 expression may serve as an indicator of treatment outcomes of MG.

Although the prognosis of MG with proper treatment is generally satisfactory (de Meel et al., 2015), our understanding of its prognostic factors is lacking (Mantegazza et al., 2003). It has been reported that the time of diagnosis from the onset is a key factor affecting treatment outcomes (Mao et al., 2010), indicating the importance of early diagnosis. Antibodies screening and observation of typical clinical features are most frequently used in the diagnosis of MG. However, diagnosis of MG can be difficult due to the difficulties in distinguishing MG symptoms from other neurological disorders and normal variants (Katalin et al., 2005). In our study, ROC curve analysis showed that downregulated GAS5 expression in PBMC effectively distinguished MG patients from healthy controls. Therefore, detection of GAS5 expression may be performed to assist the diagnosis of MG.

The pro‐inflammatory environment plays a pivotal role in the onset and development of MG (Berrih‐Aknin & Le Panse, 2014). As an anti‐inflammatory factor (Ip et al., 2017), IL‐10 was significantly inhibited in patients with MG (Sheng & Soliven, 2016). Consistently, our study also observed significantly downregulated IL‐10 mRNA expression in MG patients than in healthy controls. In cancer biology, it has been reported that GAS5 expression was negatively correlated with IL‐10 expression, and GAS5 may inhibit tumor growth by inhibiting IL‐10 secretion. Besides, a negative correlation of GAS‐5 with IL‐10 was also observed in osteoarthritis (Zhang et al., 2021). However, in our study, we observed that GAS5 expression in PBMCs was positively correlated with IL‐10 mRNA in both healthy controls and MG patients. In addition, GAS5 overexpression also significantly upregulated IL‐10 expression at the protein level in PBMCs of both MG patients and healthy controls. Therefore, GAS5 may regulate IL‐10 expression in different tissues and/or under different pathological conditions. It also suggests that GAS5 may upregulate IL‐10 expression in MG to improve disease conditions. We observed that the enhancing effect of GAS5 overexpression on IL‐10 is more significant in two cases of PBMCs derived from MG patients than in PBMCs derived from healthy controls, possibly due to the low basic IL‐10 level in PBMCs of MG patients. However, the mechanism that mediates the interaction between GAS5 and IL‐10 is unclear. Future studies are needed to explore the possible mechanisms.

In conclusion, GAS5 is downregulated in PBMCs of MG patients. GAS5 may upregulate IL‐10 expression to improve MG conditions. However, further clinical studies are still needed to confirm our conclusions.

## CONFLICT OF INTEREST

The authors declare no conflict of interest.

## AUTHOR CONTRIBUTIONS

Yangping Huang conceived and designed the study. Shuangshuang Peng carried out data interpretation and wrote the manuscript, and Yangping Huang revised the manuscript. Both authors read and approved the final manuscript.

### PEER REVIEW

The peer review history for this article is available at https://publons.com/publon/10.1002/brb3.2457

## Data Availability

Data are available from the corresponding author upon reasonable request.
